# The declining mental health of the young and the global disappearance of the unhappiness hump shape in age

**DOI:** 10.1371/journal.pone.0327858

**Published:** 2025-08-27

**Authors:** David G. Blanchflower, Alex Bryson, Xiaowei Xu

**Affiliations:** 1 Department of Economics, Dartmouth College, Hanover, New Hampshire, United States of America; 2 Adam Smith Business School, University of Glasgow, Glasgow, Scotland,; 3 National Bureau of Economic Research, Cambridge, Massachusetts, United States of America; 4 Social Research Institute, University College London, London, United Kingdom; 5 Institute for Fiscal Studies, London, United Kingdom; Niigata University, JAPAN

## Abstract

Across many studies subjective well-being has followed a U-shape in age, declining until people reach middle-age, only to rebound subsequently. Ill-being has followed a mirror-imaged hump-shape. Using graphical and regression analyses of repeat cross-sectional micro-data from the United States and the United Kingdom, we show this empirical regularity has been replaced by a monotonic decrease in ill-being by age. The reason for the change is the deterioration in young people’s mental health both absolutely and relative to older people. Pooling Global Minds data across 44 countries, including the United States and the United Kingdom, over the period 2020–2025 we confirm that ill-being is no longer hump-shaped in age but now decreases in age. JEL Codes: I31; I38

## 1. Introduction

The fact that wellbeing declines with age until middle age, then rebounds again later in life, is a key empirical regularity in the wellbeing literature. This U-shape in wellbeing by age, first described in 2008 [1], has since been replicated more than 600 times across countries and time [2]. The mid-life troughs seem to be similar in developed and developing countries at around age 50 after which well-being rises [3]. The U-shape has been apparent across a whole range of wellbeing metrics including life satisfaction and happiness.

The mirror-image of this U-shape in well-being is a hump-shape in ill-being by age which is apparent for worry, stress and depression [4–6]. In the years up to around 2015 peak ill-being in mid-life coincided with deaths of despair from suicide, drug overdoses and alcohol poisoning which also peaked in mid-life [7–10], as did psychiatric admissions [11] and the taking of anti-depressants [12]. This U-shape in well-being and hump shape in ill-being has been observed in panel data [13], suggesting that it reflects wellbeing changes over the life-course rather than differences across cohorts. Other studies confirm these patterns were apparent in multiple cohorts [14]. But no longer.

Recent evidence for Australia [15], Canada [16], New Zealand [17], the UK [18], the United States [19,20], and across 167 countries [21] points to declining well-being of the young. However, these studies do not specifically assess the implications of this change for either the hump-shape in ill-being or the U-shape in well-being by age.

We contribute to the literature by showing *for the first time* that the relative rise in ill-being among young people means that unhappiness now increases monotonically over the life-course. There is no longer a hump shape in ill-being in the United States and the United Kingdom. COVID increased the rate at which ill-being rose across all age groups, but in the UK the increase in the ill-being of the young became even more pronounced. Furthermore, since COVID the decline in ill-being with age is apparent across 44 countries, including the United States and the United Kingdom, based on comparable survey evidence on distress, fear and anxiety and suicidal thoughts in the Global Minds Dataset.

In what follows we describe rising youth mental ill-health and ill-being as well as, conversely, falling well-being based on a Mental Health Quotient (MHQ) score. We variously define mental health depending on the data file used. We define terms including despair in the BRFSS and the UK Household Longitudinal Survey as well as distress, fear and anxiety, feelings of sadness, distress or hopelessness and suicidal thoughts in the Global Minds data file. The change in the age profile of poor mental health is consistent across metrics.

## 2. The impact of rising subjective ill-being

Before presenting evidence on changes in subjective ill-being by age it is worth recalling why this might be of concern to social scientists, public health academics, policymakers and society.

First, self-reported mental health is intricately linked with physical health. Those reporting higher levels of happiness live longer [22]. Anxiety and depression slow the rate at which wounds heal. Patients scoring in the top 50% of the Hospital Anxiety and Depression Scale (HADS) were four times more likely to have delayed healing than those scoring in the bottom 50% [23].

Second, deterioration in mental health is a major cause of increasing hospital admissions among young people. According to the 2022 National Healthcare Quality and Disparities Report, in the United States from 2016 to 2019, the rates of emergency department visits with a principal diagnosis related to mental health increased for ages 0–17 years, from 784.1 per 100,000 population to 869.3 per 100,000 population. A recent cross-sectional study of 198,417 female parents of US children aged 0–17 years found large declines in their self-reported mental health from 2016 to 2023 [24].- see https://www.ncbi.nlm.nih.gov/books/NBK587174/.

Third, higher rates of depression result in higher usage of anti-depressant drugs suggesting self-reports are accurate. In the United States, the percentage of adolescents and young adults prescribed anti-depressants has been rising since before COVID [25]. The authors say this change “*was driven by increased antidepressant dispensing to females and occurred despite decreased dispensing to male adolescents*.” In the United Kingdom anti-depressant prescribing to children ages 12–17 doubled between 2005 and 2017 [26].

Fourth, declining mental health has been linked to rising suicide rates, especially among the young. In the United States, suicide is the fourth leading cause of death among those age 15–29 [27]. From 2008 to 2020, the rates of death from suicide among people aged 12 and over increased 16% overall, from 14.0 per 100,000 population to 16.3 per 100,000 population. The rate for youths aged 12–17 increased by 70% from 3.7 per 100,000 population to 6.3 per 100,000 population. S1 Fig., taken from [28], shows rising suicide rates for the young in the United States. Male rates are markedly higher than female rates, but both are on an upward trend since around 2010. Recent evidence from the European Commission has shown a rise in suicide rates of youngsters ages 15–19 in twelve EU countries between 2011 and 2022 but falls elsewhere (https://ec.europa.eu/eurostat/databrowser/view/tps00202/default/table?lang=en). Rates per 100,000 were as follows.

**Table:** 

	2011	2022
Austria	6.10	7.90
Croatia	3.69	7.39
Denmark	3.63	4.05
Germany	4.30	4.50
Iceland	4.31	8.57
Italy	2.06	2.74
Netherlands	3.99	6.35
Poland	8.06	8.32
Portugal	1.42	3.41
Spain	1.99	2.94
Switzerland	5.56	6.81
Türkiye	2.25	6.65

Fifth, deteriorating mental health is a major contributor to school absenteeism and school learning difficulties more broadly, impacting human capital investments for the next generation. In the United States, there has been a dramatic rise in chronic absenteeism defined as students missing 10 percent or more of the school year, up from 17.6% in 2017 to 29.6% in 2021 [29]. Sixty-nine percent of high school teachers in the United States in a Pew survey [30] in 2024 noted that anxiety and depression in their school was a major problem and 61% said the same about chronic absenteeism. In the United Kingdom, children aged 8−16 with a probable mental disorder were seven times more likely to have missed 15 days of school in 2022 than children without a mental disorder (https://digital.nhs.uk/data-and-information/publications/statistical/mental-health-of-children-and-young-people-in-england/2023-wave-4-follow-up).

Finally, poor mental health increasingly contributes to non-participation in the labor market. Since the pandemic, in the United Kingdom 62,000 more young people have become economically inactive, an increase of 2% [31]. Between 2019 and 2022 there was a 29% increase in economic inactivity among those aged 16–24 and a 42% increase among those aged 25–34 years. Among these age groups, the largest overall increase in people with long-term sickness was due to mental illness, which rose by around 20,000 (a 24% increase).

For all these reasons it is important to map trends in subjective ill-being over time, and by age and sex, in the United States, the United Kingdom and elsewhere.

## 3 Data and estimation

For the United States we analyze publicly available individual-level data from the Behavioral Risk Factor Surveillance System (BRFSS) conducted by the Centers for Disease Control (CDC). The BRFSS is the United States’ premier system of health-related telephone surveys that collect data regarding health-related risk behaviors, chronic health conditions, and use of preventive services. Established in 1984 with 15 states, BRFSS now collects data in all 50 states as well as the District of Columbia and three U.S. territories. It consists of more than 400,000 adult interviews each year, making it the largest continuously conducted health survey system in the world. We use data from 1993 through 2024. The data for 2024 come from the 2023 survey which, alongside the 408,012 observations in 2023, also included observations in January (n = 21,755) and February (n = 3,520) 2024. In earlier years there is also similar overlap and so, for example, 2022 includes data from the 2022 survey for that year and some additional observations from the 2021 survey, and so on.

Following Blanchflower and Oswald [32] our dependent variable is *despair*, a measure based on those who gave the answer 30 to the following question:

Q1. “*Now thinking about your mental health, which includes stress, depression, and problems with emotions, for how many days during the past 30 days was your mental health not good?*”

Both the median and the mode are zero. On average between 1993 and 2014, 66.6% of respondents reported zero days; 84.7% reported 5 or fewer; 94.3% reported 20 or fewer days while 4.8% reported exactly 30 (n = 6,182,569). In the years 2015–2024, 62.1% of respondents reported zero days; 80.0% reported 5 or fewer; 90.6% reported 20 or fewer days while 6.2% reported exactly 30 (n = 3,899,720). Overall, the (weighted) incidence of despair nearly doubled from 3.7% (N = 100,090) in 1993 to 6.7% in 2023/24 (N = 450,264). It rose from 2.9% in 1993 to 8% in 2023 for those under age 25.

For the United Kingdom, we examine data from the UK Household Longitudinal Survey (UKHLS), a household panel study, from 2009–10–2022–23. The survey has an overlapping wave structure, where each wave of fieldwork spans two overlapping calendar years (2009–10, 2010–11 and so on). Our dependent variable comes from the General Health Questionnaire mental health index (GHQ-12) scored on a scale of 0–36 (Likert scale) where a higher score denotes poorer mental health. In 2009–10 the median was 10 and in 2022–23 the median was 11 among those aged 18–70. We classify people with a score above 23 as being in ‘despair’, to align with the proportion of people in despair in the United States across the 2009–2014 period (5.3%). Approximately 4.6% of all respondents were classed as being in despair in 2009–10, rising to 8.1% in 2022–2023. The distribution of GHQ scores is smooth around 23, as shown in S2 Fig, so our results are not sensitive to the precise choice of cut-off.

We also make use of individual data from the United Kingdom Annual Population Surveys, 2012–2021 which contain questions on anxiety. The 11-step scale running from 0 to 10 records responses to the question:

*Q2. “On a scale where nought is ‘not at all anxious’ and 10 is ‘completely anxious’, overall, how anxious did you feel yesterday?”*

Finally, we examine data from the Global Minds Project [33] for the years 2020−2025. We restrict analysis to 44 countries with at least 10,000 observations giving us 1,702,498 (84% of the overall sample of 1,912,156). S1 Table. sets out the number of observations by country across the six years. These data are collected online. The survey takes around 15 minutes to complete. Information is collected on mental wellbeing and used to construct a Mental Health Quotient (MHQ) assessment of people’s cognitive and emotional capabilities, calculated on a 300-point scale running from −100 to +200 where higher scores denote better mental health. The MHQ contains six domains: overall hand function; activities of daily living; work performance; pain; aesthetics and satisfaction. Scores in the normal healthy range span from 0 to 200 [34]. The mean overall MHQ for the full sample over the years 2020−2025 is 68 (SD 73). Scores are classified into six groups and respondents receive a report with their score. The overall 2020−2025 percentages are in round parentheses and the percentages for those aged under-25 in square parentheses.

**Table:** 

Thriving	150–200	(13.9%)	[3.6%]
Succeeding	100–149	(26.7%)	[13.3%]
Managing	50–99	(20.4%)	[17.9%]
Enduring	>0–49	(13.5%)	[16.7%]
Struggling	−50 to <0	(19.7%)	[35.0%]
Distressed	−100 to <−50	(5.6%)	[13.4%]

Overall, 25% of respondents and 48% of those aged under-25, were classified as clinically at-risk, respectively, with negative scores. Twenty-one percent of females aged 25-and-over had negative scores versus 17% of males in the same age range. Among those aged under-25 the percentages were 53% for females and 41% for males. One-in-twenty (5.6%) of the sample were “distressed” – scoring less than −50 on the MHQ – but the incidence of distress was twice as high among under-25s (13.4%).

In analyzing the data we focus on those aged 18–74 years across 44 countries. We present results for five measures of mental ill-health:

Q3. *Mental Health Quotient*. Positive affect variable used by the GM project, higher score implies higher well-being. Mean = 68.

Q4. *Distressed*, a (1,0) dummy to identify those distressed based on MHQ score from −100 to <−50. Mean = .13.

Q5. *Feelings of sadness, distress or hopelessness* - “Experiencing overwhelming feelings of unhappiness, sorrow and hopelessness, or having spells of uncontrollable crying” *mean = 4.45.*

Q6. *Fear and anxiety -* “Being scared or worried and experiencing feelings and sensations of nervousness or panic in your mind or body” mean = 5.24.

Q7. *Suicidal –* “Thinking or feeling like you want to kill or physically harm yourself’ mean = 2.64.

Variables Q3 and Q5-Q7 form part of the MHQ score and are scored from 1–9 where 1 = ’never causes me any problems’: 5 ‘sometimes causes me difficulties or distress but I can manage’ and 9=’has a constant and severe impact on my ability to function’. In regression analyses presented below we control for country, year and month of interview, gender, education and labor force status, as well as age.

We report regression analyses using Ordinary Least Squares (OLS) estimation. Control variables are presented in each table, along with unweighted estimation sample sizes and the adjusted r-squared for the model. In addition to coefficients, we report T-statistics in parentheses.

## 4 Results

We depict trends in ill-being graphically, first for the United States then the United Kingdom before estimating regressions to capture the independent correlation between age and ill-being and how this has changed over time. Finally, we present both graphical and regression analyses to examine the association between age and ill-being across the world.

### 4.1 Trends in Subjective Ill-being by Age in the United States

Table 1 reports the proportion of respondents to the BRFSS who report being in despair in the years since 1993, overall and for the young by gender. The rise for the young in general and especially young women is notable.

**Table 1:** Despair in the USA, 1993–2024, BRFSS (%).

	All	Females age < 25	Males age < 25
1993	3.64	3.21	2.50
1994	4.02	4.67	2.83
1995	4.26	4.26	2.91
1996	4.15	4.24	2.42
1997	4.34	4.34	3.36
1998	4.38	5.30	2.87
1999	4.35	3.68	3.16
2000	4.44	5.03	3.58
2001	4.85	5.12	3.46
2002	4.55	4.54	4.11
2003	4.90	5.24	4.19
2004	4.95	5.41	4.10
2005	4.70	5.68	3.35
2006	4.87	5.82	3.64
2007	4.85	4.77	3.82
2008	4.98	4.96	3.07
2009	5.14	5.63	2.99
2010	5.12	4.77	3.35
2011	5.66	5.11	3.51
2012	5.82	5.91	3.98
2013	5.59	6.08	3.52
2014	5.62	5.19	3.93
2015	5.48	5.53	3.99
2016	5.70	6.51	3.88
2017	5.94	6.90	5.35
2018	6.18	8.91	5.33
2019	6.25	8.70	5.76
2020	5.99	8.24	5.09
2021	6.51	9.68	6.37
2022	7.00	10.82	7.25
2023/4	6.63	9.32	6.63

Notes: “Despair” is set to 1 (zero otherwise) if the answer to this question was all 30 days. Q1. “*Now thinking about your mental health, which includes stress, depression, and problems with emotions, for how many days during the past 30 days was your mental health not good?*”

**Fig 1:**
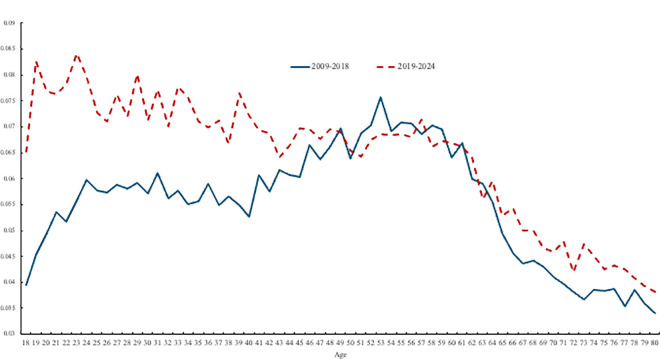
Despair in the USA, 1993-2024.

Fig 1 plots trends in despair by agein the United States for two periods - 2009-2018 (the blue line) and 2019-2024 (the red dotted line). It is apparent that the hump-shape in despair by age in the first period has been replaced by despair declining in age due to a rise in the rate of despair among younger people.

**Fig 2:**
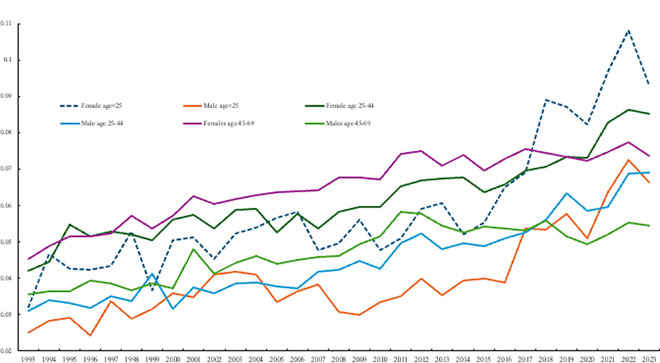
Despair by age, BRFSS.

Fig 2 plots these data for the United States going back to the early 1990s for men and women by age group, separately.

For both genders, levels of despair in 2009 were highest among the oldest age group (45–70) and higher for the middle-aged (25–44) than the young (18–24). However, the percentage of young people in despair rose rapidly over this period, more than doubling for men (from 3.0% to 6.6%) and almost doubling for women (5.6% to 9.3%). Despair also rose among the middle-aged, but less rapidly (from 6.0% to 8.5% for women and from 4.5% to 6.9% for men), while the percentage of older men and women in despair remained roughly constant over the period. As result, by 2023/24 relative levels of despair across age groups were reversed for women, with the youngest age group having the highest levels of despair, and the oldest age group the lowest. For men, the level of despair was similar for the youngest and middle-aged groups, and lowest for the oldest age group.

**Fig 3:**
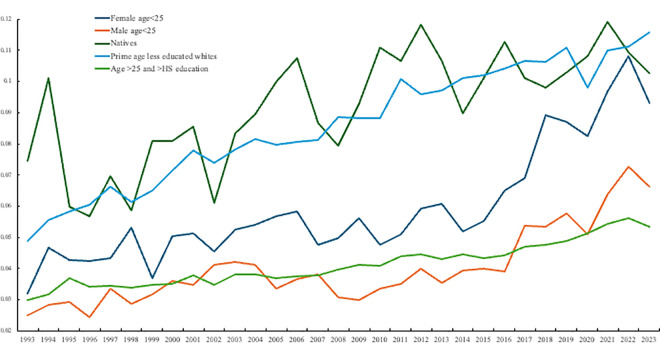
Despair by age, 1993–2023.

Fig 3 does the same, but also plots change for natives, less educated prime age whites, and older people with more than high school education. The concern in the ‘deaths of despair’ literature was the problem faced by prime-aged (35–54-year-olds) whites with a high school diploma or less [7]. What Figs 2 and 3 illustrate is that, whilst we should continue to be concerned about these groups in the population since their levels of despair remain high, since around 2014 the rate of despair has grown most quickly among the young, especially females under age 25, such that by the end of the period their despair levels are on a par with those who were the focus of Case and Deaton’s [7] work. By 2022 the despair levels of young females matched those of natives and prime age less educated whites. As noted above we have already started to see a rise in suicide rates in the young but, to this point, there is no evidence of rising drug overdose deaths among the young [9].

These trends have resulted in a very different relationship between age and ill-being over time in the United States, as indicated in Fig 1. Between 2009 and 2018, despair is hump-shaped in age, very much in accordance with the literature discussed in the introduction. The rapid rise in despair before the age of 45, and especially before the mid-20s, has fundamentally changed the lifecycle profile of despair, such that the hump-shape is no longer apparent between 2019 and 2023 (dotted line in Fig 1). Despair rose the most for the youngest group but also rose for those up to age 45; it remained unchanged for those aged over-45. Despair is now declining monotonically in age.

### 4.2. Trends in Subjective Ill-being by Age in the United Kingdom

In this section we present similar trends for the United Kingdom, building on work first reported in Banks and Xu [18] using data from the UK Household Longitudinal Survey (UKHLS) from 2009−2021, supplementing it with analyses of anxiety using the Annual Population Survey from 2012 to 2021. For brevity, we refer to the first year of each wave in the UKHLS, with 2009 referring to the 2009−10 wave, 2010 referring to the 2010−11 wave and so on.

**Fig 4:**
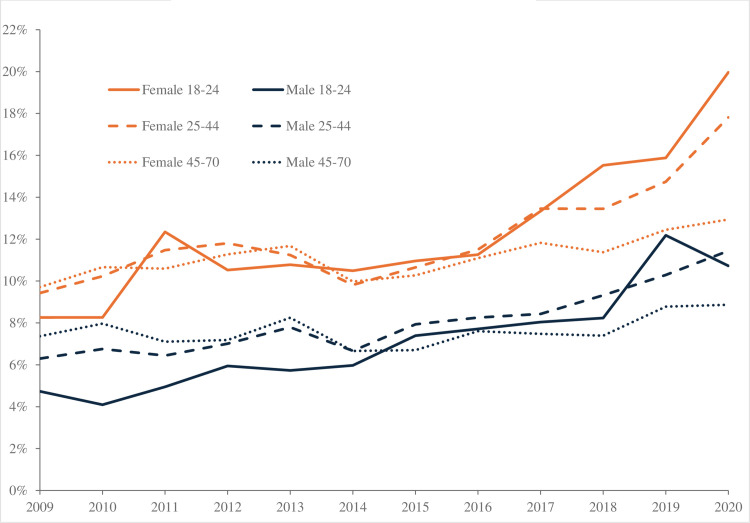
Despair by age UK, UKHLS, 2009–2021.

Trends in despair across age groups are similar to those in the United States, as shown in Fig 4. In 2009, both men and women aged 18–24 were less likely to be in despair than older age groups. Among men under 25, despair more than doubled between 2009 and 2021 (from 2.3% to 6.4%), as it did in the United States. The percentage of young women in despair rose even more sharply, from 4.4% to 12.7%, with most of the increase coming after 2016. Levels of despair also increased for both men and women in older age groups, but the rise for the middle-aged group was smaller than for the youngest, and the rise for the oldest age group was smaller still.

**Fig 5:**
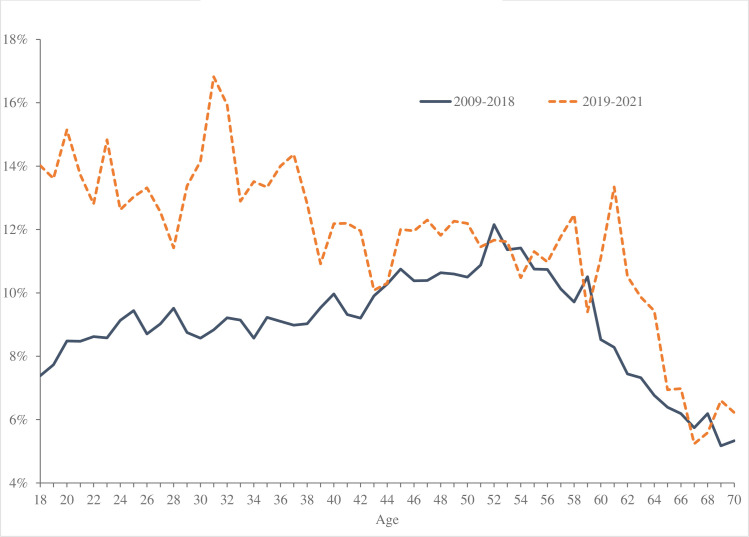
Age profile in despair UK, UKHLS.

As a result, the hump-shape in despair by age, notable in the earlier period (2009–2018), has disappeared in the later period 2019–2021. It has been replaced by a profile of despair that is declining in age, as shown in Fig 5. As in the case of the United States, the age-profile of despair did not change markedly among those in their late 40s and older, but levels of despair rose strongly among those below their mid-40s, especially among the youngest.

**Fig 6:**
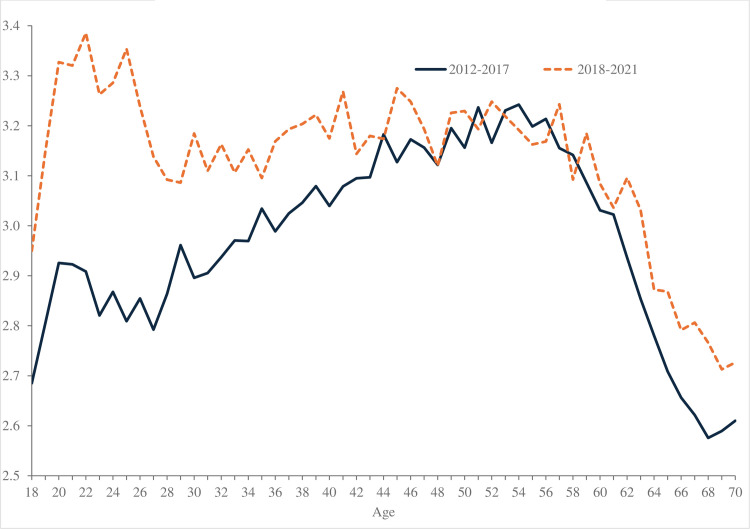
Age profile in anxiety UK, Annual Population Survey, 2012–2021.

The disappearance of the hump-shape is also seen for anxiety, shown in Fig 6 using APS data. The rapid rise in anxiety among the young means that the hump-shaped profile observed in 2012–2017 is no longer seen in 2018–2021. Anxiety now broadly declines with age in the United Kingdom. Table 2 reports annual means in the same anxiety data for the UK by age and ethnicity. It shows the rise in anxiety overall from 2011 to 2021, as well as notable rises for all young females under age 25.

**Table 2:** Mean Scores in 11-step anxiety in UK, 2012–2021 (weighted), 2011–2021.

	All	Male <25	Female <25 White	Whites Females <25	Whites	Blacks	Asians
2011	3.13*						
2012	3.03	2.75	2.99	2.95	3.01	3.18	3.20
2013	2.95	2.60	2.95	2.90	2.92	3.14	3.08
2014	2.89	2.54	2.86	2.84	2.87	2.94	2.95
2015	2.85	2.53	2.85	2.84	2.84	2.79	2.92
2016	2.89	2.66	3.17	3.21	2.88	2.85	2.91
2017	2.91	2.71	3.27	3.30	2.89	3.06	2.95
2018	2.85	2.67	3.18	3.20	2.85	2.74	2.80
2019	2.94	2.71	3.57	3.66	2.93	2.85	2.85
2020	3.33	2.96	3.80	3.80	3.32	3.36	3.33
2021	3.12	2.92	3.82	3.86	3.13	2.95	2.94
2022	3.23*						

Notes: * estimates from Personal well-being in the UK: April 2022 to March 2023, ONS, Nov 13, 2024.

Source: UK Annual Population Surveys (weighted).

## 5. Econometric analysis

Table 3 presents despair equations for the United States (Panel A) and the United Kingdom (Panel B) for men and women separately using data from the BRFSS and the UKHLS respectively. Equations include age dummies plus linear year trends in the first column. In column 2 we add age group interactions with COVID dummies where COVID is defined as years 2020 and 2021.

**Table 3:** Despair OLS regressions in the USA and the UK ages 18–70.

A) USA				
	Men		Women	
18–24 * Year	.0029 (18.23)	.0029 (11.73)	.0048 (24.83)	.0045 (15.24)
25–44 * Year	.0018 (21.06)	.0019 (14.87)	.0022 (25.04)	.0017 (12.47)
45–70 * Year	−.0002 (3.10)	−.0001 (0.62)	.0003 (4.36)	.0002 (2.17)
18–24 * Covid		.0002 (0.09)		.0029 (1.23)
25–44 * Covid		.0018 (1.78)		.0056 (4.97)
45–70 * Covid		−.0014 (1.88)		.0007 (0.98)
25–44	2.3459 (6.45)	1.9541 (6.45)	5.2217 (12.17)	5.7018 (8.66)
45–70	6.2512 (18.19)	5.9619 (11.25)	9.1623 (22.44)	8.7373 (13.97)
Constant	5.8347	5.7999	−9.6175	−9.0605
Adjusted R^2^	.0005	.0005	.0006	.0006
N	1,899,520	1,899,520	2,335,810	2,335,815
B) UK				
	Men		Women	
18–24 * Year	.0061 (11.21)	.0046 (6.60)	.0079 (13.26)	.0059 (7.74)
25–44 * Year	.0039 (11.75)	.0030 (7.09)	.0049 (14.13)	.0032 (7.23)
45–70 * Year	.0007 (2.84)	−.0003 (0.75)	.0019 (6.61)	.0012 (3.28)
18–24 * Covid		.0249 (3.57)		.0311 (4.09)
25–44 * Covid		.0160 (3.66)		.0301 (6.52)
45–70 * Covid		.0161 (4.74)		.0111 (3.03)
25–44	.0235 (5.35	.0210 (4.37)	.0173 (3.60)	.0163 (3.10)
45–70	.0393 (9.22)	.0375 (8.02)	.0274 (5.88)	.0226 (4.41)
Constant	.0316	.0376	.0714	.0791
Adjusted R^2^	.0018	.0021	.0022	.0025
N	158,899	158,899	203,175	203,175

T-statistics in parentheses.

Source: BRFSS 1993–2022 and UKHLS 2009–2022.

In the first column we see that despair increased among men and women aged under-45 in the USA and the UK, but the increase is most pronounced among those aged under-25. In column 2 we supplement the year interactions with interaction terms to establish whether the rate of change changed with the onset of COVID. This shows that in the UK, the rate of increase in despair among under-25s increased during COVID even when compared to the underlying trend captured by the linear trend. This was also the case for older age groups, though the increase during COVID was less pronounced. In the United States, the increase in despair during the COVID period is largely confined to middle-aged (25–44) women. 

In Tables 4–6 we delve further into the correlates of despair in the United States and the United Kingdom and how they have changed over time using the micro data from BRFSS for the USA (1993–2024) and anxiety in the UK using the APS (2012–2021). There are many similarities with anxiety rising among the young and females, and especially young females. The econometric analysis confirms the time series evidence in the figures and charts presented above.

**Table 4:** OLS Despair equations for all in the United States, 1993–2024.

	1993–1999	2000–2010	2011–2015	2016–2024
Female	.0141 (30.53)	.0137 (54.65)	.0114 (38.24)	.0145 (57.13)
25–34	.0071 (7.70)	.0093 (14.06)	.0129 (15.77)	.0025 (3.67)
35–44	.0125 (13.63)	.0116 (17.97)	.0130 (16.06)	−.0016 (2.44)
45–54	.0136 (14.12)	.0145 (22.54)	.0127 (16.22)	−.0097 (14.74)
55–64	−.0019 (1.80)	−.0003 (0.50)	.0002 (0.30)	−.0251 (38.73)
65–74	−.0117 (9.27)	−.0193 (26.67)	−.0171 (20.21)	−.0386 (55.42)
75+	−.0110 (7.85)	−.0259 (33.85)	−.0269 (30.10)	−.0499 (67.19)
_cons	.0260	.0498	.0497	.0539
Adjusted R^2^	.0332	.0544	.0585	.0469
N	873,911	3,412,943	2,317,981	3,451,071

Notes: excluded categories under-25. Equations also include samples and controls for Puerto Rico, Guam and Virgin Islands and education, race, state, year and labor force status, results not reported. T-statistics in parentheses. Source: BRFSS 1993–2024.

**Table 5:** Despair equations for under-25s in the United States, 1993–2024.

	1993–1999	2000–2010	2011–2015	2016–2024
Female	.0158 (11.42)	.0196 (18.04)	.0199 (16.24)	.0353 (31.10)
19	.0112 (4.11)	.0123 (5.89)	.0148 (6.19)	.0142 (6.42)
20	.0165 (5.95)	.0206 (9.66)	.0201 (8.29)	.0162 (7.26)
21	.0151 (5.65)	.0243 (11.77)	.0231 (9.66)	.0168 (7.57)
22	.0167 (6.18)	.0271 (12.95)	.0258 (10.58)	.0176 (7.80)
23	.0227 (8.41)	.0297 (14.24)	.0284 (11.53)	.0232 (10.13)
24	.0216 (7.96)	.0284 (13.59)	.0297 (11.96)	.0222 (9.61)
_cons	−.0040	.0086	.0033	.0259
Adjusted R^2^	.0122	.0188	.0200	.0200
N	80,572	167,400	120,947	205,618

Notes: excluded categories age 18. Equations also include samples and controls for Puerto Rico, Guam and Virgin Islands plus education, year, race, state and labor force status dummies. T-statistics in parentheses.

Source: BRFSS 1993–2024.

**Table 6:** Anxiety in the UK, 2012–2021.

	All Ages		Age < 25	
	2012–2017	2018–2021	2012–2017	2018–2021
20–24	.3057 (10.13)	.4596 (8.36)		
25–29	.3947 (12.51)	.4442 (7.81)		
30–34	.5078 (16.21)	.4415 (7.85)		
35–39	.6048 (19.34)	.4777 (8.52)		
40–44	.7044 (22.67)	.5158 (9.18)		
45–49	.7784 (25.20)	.5445 (9.75)		
50–54	.8316 (26.95)	.5644 (10.16)		
55–59	.6994 (22.60)	.4443 (8.01)		
60–64	.2920 (9.38)	.1796 (3.23)		
65–69	−.1349 (4.30)	−.3164 (5.64)		
70–74	−.0099 (0.25)	−.1178 (1.80)		
75+	−.0239 (0.59)	−.1316 (1.99)		
17			.1478 (1.85)	.3725 (2.58)
18			.2239 (2.92)	.6059 (4.30)
19			.3204 (4.31)	.6505 (4.87)
20			.4289 (5.93)	.8424 (6.44)
21			.4917 (6.78)	.8872 (6.81)
22			.5162 (7.01)	.9675 (7.31)
23			.4796 (6.48)	.8440 (6.31)
24			.5531 (7.47)	.8846 (6.62)
Female	.2714 (45.67)	.4063 (43.15)	.3707 (5.01)	.6637 (14.71)
_con	2.8960	3.102	2.1807	2.3031
Adjusted R^2^	.0158	.0188	.0123	.0266
N	960,297	378,582	52,258	15,553

Notes: excluded 16–19 and 16 in columns 3–4; controls include education, labor force status, region and race dummies. Source: UK Annual Population Surveys

In Table 4 we run four sets of OLS regressions for all age groups in the United States with the dependent variable despair (as in 30/30 days were bad mental health days). Estimates are presented for 1993–1999, 2000–2010, 2011–2015 and 2016–2024 in columns 1–4 respectively. Sample sizes are 874,000 in the first period and 2.3–3.5 million in the remaining three. In addition to age dummies the models include controls for gender, education, race, labor force status, state and year. The changing age pattern of despair is confirmed here as the age coefficients in the final column, compared with the excluded of 18–24-year-olds, no longer peak in midlife as they do in the first three columns for earlier periods. The differences in the age coefficients across the time-periods are statistically significant and are consistent with the change in the age profile in despair presented in Fig 3. There is no longer a hump-shape in despair in age.

Table 5 reruns the analyses in Table 4 but limits the sample to those aged under-25. It includes the same controls as in Table 4 but now also includes single year of age dummies from 19–24 which are compared with the excluded category which is age 18. Within this age group despair rises with age. Of note here is the marked rise in the size of the female coefficient, especially in the final period.

Table 6 estimates similar equations to those for the United States above with the anxiety variable from the APS for the United Kingdom. The sample is split into two time periods, 2012–2017 (columns 1 and 3) and 2018–2021 (columns 2 and 4). In the first two columns, which are for all ages, we see age effects shift: anxiety rises markedly among the under-25s in the second period, and to some extent among those aged 25–29 years, but it falls for all other age groups as is seen in Fig 6. The female coefficient rises in the second period. Columns 3 and 4 relate to those age under 25. Anxiety is rising with age among the young, and this age gradient is much more pronounced in the second period. The US and UK results are broadly consistent.

## 6. Trends in Subjective Ill-being by Age in the Rest of the World

To establish whether this change in the age profile of subjective ill-being is apparent elsewhere in the world we turn to the Global Mind Project described earlier. We examine data for 2020–2025 for 44 countries with at least 10,000 observations pooled over the years with broad coverage across the world. The data have coverage across six broad areas:

1) Middle East (3) – Israel, Jordan and Saudi Arabia.
2) Africa (10) – Algeria, Angola, Egypt, Kenya, Morocco, Mozambique, Nigeria, South Africa, Tunisia and Yemen.
3) Latin America (15) – Argentina, Bolivia, Brazil, Chile, Colombia, Ecuador, El Salvador, Guatemala, Honduras, Mexico, Nicaragua, Paraguay, Peru, Uruguay and Venezuela.
4) Asia (5) – Bangladesh, India, Iraq, Pakistan and the Philippines.
5) English speaking countries (6) Australia, Canada, Ireland, New Zealand, UK and USA.
6) Europe (5) – Belgium, France, Germany, Italy and Spain.

Table 7 shows the percent ‘distressed’ falls more or less linearly with age in all 44 countries (which include the United States and the United Kingdom): there is no indication of a hump shape in ill-being with age. The sample is restricted to those ages 18–74. If we focus on differences by sex among the under-25s we find the mental health of women is worse than that of men in all 44 countries for despair and suicidality (Table 8).

**Table 7:** Age distribution for distress, global minds 2020–2025 age 18–74.

	Algeria	Angola	Argentina	Australia	Bangladesh	Belgium	Bolivia	Brazil	Canada	Chile	Colombia	Ecuador
18–24	.105	.098	.139	.141	.136	.114	.172	.143	.115	.169	.148	.178
25–34	.057	.053	.069	.098	.088	.035	.093	.118	.077	.097	.081	.069
35–44	.037	.036	.040	.079	.040	.032	.038	.093	.055	.067	.047	.045
45–54	.026	.040	.029	.061	.020	.047	.022	.070	.053	.041	.033	.024
55–64	.014	.021	.017	.048	.010	.023	.013	.041	.026	.025	.016	.010
65–74	.010	.028	.011	.016	.010	.011	.008	.021	.011	.013	.013	.008
All	.047	.064	.043	.073	.064	.029	.071	.061	.049	.041	.082	.066
N	56,208	15,704	81,180	25,593	12,119	8,955	15,118	14,285	33,057	17,566	56497	14,792
	Egypt	El Salvador	France	Germany	Guatemala	Honduras	India	Iraq	Ireland	Israel	Italy	Jordan
18–24	.125	.146	.168	.123	.145	.150	.158	.137	.128	.069	.053	.103
25–34	.081	.077	.065	.060	.078	.061	.128	.068	.099	.065	.012	.062
35–44	.044	.034	.051	.047	.040	.035	.072	.037	.096	.036	.033	.033
45–54	.028	.017	.037	.051	.020	.016	.030	.018	.049	.020	.036	.020
55–64	.016	.013	.020	.038	.014	.015	.017	.015	.031	.014	.021	.011
65–74	.011	.006	.012	.017	.011	.011	.010	.010	.018	.009	.010	.015
All	.056	.047	.051	.049	.052	.048	.088	.068	.050	.028	.019	.040
N	96,908	11,673	37,590	26,216	16,746	11,092	206,413	33,620	10,011	15,868	27,716	31,944
	Kenya	Mexico	Morocco	Mozambique	NZ	Nicaragua	Nigeria	Pakistan	Paraguay	Peru	Philippines	SArabia
18–24	.080	.177	.104	.081	.159	.162	.083	.132	.135	.174	.058	.140
25–34	.060	.100	.057	.041	.106	.071	.035	.100	.068	.101	.067	.067
35–44	.040	.058	.037	.036	.044	.037	.020	.039	.029	.044	.031	.042
45–54	.023	.032	.024	.030	.053	.017	.013	.020	.023	.016	.015	.025
55–64	.010	.016	.018	.021	.024	.021	.012	.011	.017	.013	.009	.020
65–74	.008	.011	.010	.023	.011	.017	.013	.011	.012	.008	.009	.014
All	.042	.098	.043	.048	.052	.058	.026	.054	.051	.082	.028	.055
N	10,510	97,144	36,239	13,299	11,227	10,917	30,528	49,688	14,749	27,794	30,185	16,424
	SAfrica	Spain	Tunisia	UK	USA	Uruguay	Venezuela	Yemen	All			
18–24	.157	.136	.093	.156	.104	.132	.104	.071	.137			
25–34	.101	.084	.060	.112	.066	.054	.055	.049	.082			
35–44	.083	.072	.040	.100	.061	.032	.033	.031	.048			
45–54	.064	.055	.026	.088	.053	.033	.016	.020	.033			
55–64	.033	.033	.015	.063	.027	.015	.011	.017	.022			
65–74	.012	.011	.007	.025	.011	.014	.010	.009	.009			
All	.076	.070	.035	.086	.046	.028	.039	.047	.060			
N	38,115	47,956	22,664	59,103	95,754	12,779	69,695	34,659	1,605.760			

**Table 8:** Feelings of distress by gender for the young age < 25 by 44 countries, 2020–2025.

	Female	Male		Female	Male
Algeria	.109	.097	Italy	.060	.041
Angola	.130	.050	Jordan	.111	.085
Argentina	.167	.098	Kenya	.085	.072
Australia	.152	.121	Mexico	.206	.130
Bangladesh	.156	.086	Morocco	.115	.082
Belgium	.164	.049	Mozambique	.105	.046
Bolivia	.201	.115	New Zealand	.173	.142
Brazil	.164	.112	Nicaragua	.203	.096
Canada	.121	.106	Nigeria	.091	.063
Chile	.207	.121	Pakistan	.143	.097
Colombia	.177	.101	Paraguay	.171	.086
Ecuador	.201	.135	Peru	.199	.131
Egypt	.130	.116	Philippines	.068	.041
El Salvador	.180	.089	Saudi Arabia	.148	.118
France	.184	.133	South Africa	.177	.115
Germany	.141	.094	Spain	.154	.103
Guatemala	.172	.105	Tunisia	.098	.081
Honduras	.172	.113	United Kingdom	.167	.140
India	.177	.127	United States	.106	.100
Iraq	.148	.112	Uruguay	.167	.084
Ireland	.150	.102	Venezuela	.131	.068
Israel	.080	.049	Yemen	.083	.056

OLS regression analyses for the five illbeing metrics described earlier show that ill-being, however defined, declines with age in the period 2020–2025 (Table 9). The MHQ score rises with age – a higher score is better. These estimates control for country, gender, education, labor force status, and year. But they also control for month, day and time of survey, something that can play an important role in way respondents answer such questions [35]. Adding controls does not change the story. Ill-being declines with age. At a suggestion of a referee in Table 9 we run separate distressed equations for the Middle East, Asia, Latin America and Africa plus for the English-speaking countries with the Europe. The results are very similar, the probability of being depressed declines in age in all. Females in each case have a higher incidence of distress.

**Table 9:** Negative affect regressions from Global Minds Database, 2020–2025, age 18–74.

*a) All countries*					
	MHQ score	Distressed	Sadness, distress or hopelessness	Fear and Anxiety	Suicidal thoughts or intentions
Female	13.921 (27.13)	−.0727 (39.80)	−.1008 (4.88)	−.0169 (0.90)	−.8100 (42.69)
Male	23.888 (46.27)	−.0908 (49.39)	−.8035 (38.69)	−.5831 (30.65)	−.9541 (49.98)
25–34	12.870 (60.42)	−.0385 (50.78	−.3187 (37.19)	−.1577 (20.08)	−.6889 (87.47)
35–44	30.809 (140.09)	−.0676 (86.43)	−.9364 (105.85)	−.6160 (76.00)	−1.2631 (155.33)
45–54	47.063 (215.52)	−.0846 (108.80)	−1.4559 (165.73)	−1.0160 (126.25)	−1.5818 (195.91)
55–64	61.230 (275.92)	−.0978 (123.78)	−1.9167 (214.72)	−1.3533 (165.47)	−1.8471 (225.13)
65–74	73.074 (271.36)	−.1065 (111.09)	−2.3164 (213.85)	−1.6504 (166.31)	−2.0779 (208.71)
_cons	11.733	.1785	5.9465	6.4675	4.0716
Adjusted R^2^	.2154	.0555	.1570	.1027	.1443
N	1,605,744	1,605,744	1,605,661	1,605,687	1,605,673
*b) Distressed by Continents*					
	Middle East	Asia	Latin America	Africa	Advanced
Female	−.0381 (4.65)	−.0246 (4.82)	−.1325 (35.96)	−.0172 (4.43)	−.0861 (29.34)
Male	−.0449 (5.45)	−.0476 (9.28)	−.1632 (44.10)	−.0297 (7.60)	−.0931 (31.50)
25–34	−.0267 (7.86)	−.0243 (13.61)	−.0545 (38.35)	−.0308 (22.01)	−.0396 (23.11)
35–44	−.0509 (15.69)	−.0718 (36.29)	−.0872 (60.11)	−.0499 (35.18)	−.0534 (29.86)
45–54	−.0641 (20.23)	−.0990 (50.54)	−.1035 (72.31)	−.0625 (42.05)	−.0679 (41.44)
55–64	−.0685 (20.10)	−.1074 (51.97)	−.1138 (79.06)	−.0733 (43.43)	−.0918 (60.45)
65–74	−.0671 (15.91)	−.1115 (43.78)	−.1194 (68.82)	−.0827 (36.41)	−.1033 (58.84)
_cons	.0907	.1250	.2123	.1132	.1844
Adjusted R^2^	.0431	.0557	.0693	.0345	.0616
N	64,230	332,023	472,027	354,826	382,638

Excluded: 18–24 and other gender, includes schooling, labor force status, country and year dummies. T-statistics in parentheses.

*“Feelings of sadness, distress or hopelessness”, “Feelings of fear and anxiety”,“Thinking or feeling like you want to kill or physically harm yourself”*. 1–9 scale: 1 never causes me any problems: 5 sometimes causes me difficulties or distress but I can manage; 9 = has a constant and severe impact on my ability to function. Distressed is set to 1 if mhq < 0, zero if mhq 0–200.

Advanced countries are Europe and English-speaking countries. Distressed is set to 1 if mhq < 0, zero if mhq 0–200.

## 7. Discussion

The well-being U-shape in age and the hump-shape in ill-being has been described by one of us as ‘among the most striking, persistent patterns in social science’ [6]. This is no longer the case. Instead, subjective ill-being falls with age, in the United States, the United Kingdom and in 42 other countries. In the United States and the United Kingdom, we can show that this change has come about because the mental health of the young has deteriorated compared to that of older people. It seems reasonable to infer that the same underlying changes account for the age pattern in illbeing we observe across the world in the Global Minds data for 2020–2025.

It does not seem that the results have been driven by the pandemic alone as we show that the decline in both the US and the UK started prior to the pandemic, which simply exacerbated existing trends. There is also little evidence that the changes were driven by the financial crisis. A referee has suggested to us that the observed declines in youth wellbeing may well be because there are now different expectations of younger cohorts, perhaps the results of objectively tougher conditions on access to housing and the labor market.

What lies behind the differential rate of decline in wellbeing by age over the last decade or so? The answer is unclear, but several factors may be at play. One is the potential ‘scarring’ effect of the Great Recession on new cohorts entering the labor market – the wellbeing analogue of the scarring effects of recession on new cohorts’ subsequent labor market prospects [36,37]. These effects might arise through negative shocks to employment prospects and wage growth for new entrants to the labor market around the time of a severe economic shock, such as the Great Recession of 2008. These effects could ‘scar’ in the sense that they might persist for some time causing permanent scars rather than temporary blemishes [38].

It is possible that, because the labor market did not recover quickly after the Great Recession – as indicated by real wage stagnation – successive cohorts of new entrants may have been impacted in the years following the Great Recession shock. Of course, the Great Recession impacted the labor market prospects and household income for older people too, potentially explaining the deterioration in their wellbeing. But, as in the case of labor market scarring, effects may be felt most and persist for longest among the young. However, any Great Recession-induced decline in labor market prospects cannot account for the deterioration in adolescent mental health which occurs between age 10 and 16 – a pattern that has been observed in cross-sectional data for 43 countries and replicated using within-person analysis of the UKHLS [39,40].

Three other hypotheses might account for the marked decline in wellbeing among the young. The first is the depletion of health care resources available to treat mental health conditions. Ever since the Great Recession monies available for publicly provided health care services have been stretched. There is recognition in both the United States and the United Kingdom that mental health services are acutely underfunded [7] such that delays in access to treatment may have prolonged the duration of spells of poor mental health which, in cross-sectional data, will be evident in an increase in the ‘stock’ of individuals suffering from poor mental health.

Second, much of the recent literature on the deterioration of mental health among the young has focused on the COVID pandemic. Although it cannot account for the decline in mental health among the young going back to the period shortly after the Great Recession, it may have contributed to an increasing rate of deterioration in young people’s mental health. Our own data provide some support for this proposition since in the UK the increase in despair among the young relative to older age groups has risen since the onset of COVID.

The third hypothesis relates to the advent of smart phone technologies and the way they have impacted young people’s perceptions of themselves and their lives relative to their peers’ portrayal of their lives via social media. This new information about their lives may result in greater dissatisfaction with one’s own life, in much the same way that new information about the ‘pay gap’ between one’s own pay and that of colleagues’ generates increased pay dissatisfaction [41]. The growth in smart phone usage coincides with the rising trend in ill-being. There is a growing body of evidence suggesting that the rise in ill-being of the young is associated with the rise in the use of the internet and smartphones [21,42–45]. Some studies that randomize restricted access to smart phones identify significant improvements in self-reported wellbeing [46] leading to calls for restrictions on young people’s access to smart phone technology and in the regulation of social media content.

There is debate about whether any link between smart phone use and poor mental health is causal. However, there is a growing recent ‘natural experiment’ literature summarized by Pugno (2025) [47], suggesting that indeed the relationship between smartphone use and worsening youth mental health is indeed causal. Pugno cites studies for the US [48], the UK [49], Germany [50], Italy [51] and Spain [52] based on ‘natural experiments’, based on comparing a sample of the population that has access to social media to another very similar sample that does not. Braghieri et al [48], for example, make use of evidence on the spread of Facebook across US college campuses, while the remaining studies [49–52] make use of data on the spread of broadband by area and show these have a sizeable and negative impact on youth wellbeing, and that of young women in particular. This seems to settle the matter. See also McClean, Rausch and Haidt [53] arguing similarly that these studies establish causality. They further note that Kyung and Lee [54] find that broadband access in the US during 2013–2017 increased suicide rates.

In this paper we have mapped a change in the age-pattern of ill-being due to rising mental ill-being among the young. We have done so for the United States and the United Kingdom. We have shown for these two countries plus a further 42 countries from around the world in the years since 2020, that ill-being declines in age. There is no longer a hump-shape in ill-being by age. The question this begs is what to do about this phenomenon of a global decline in youth well-being that shows no sign of abating?

## Supporting information

S1 Fig US Suicide Rate. (TIF)

S2 Fig Density Plot for GHQ Likert Scale in the UK from UKHLS. (TIF)

S1 Table 44 country observations in the Global Minds database by year. (DOCX)
