# Supplementary figures and images for: The declining mental health of the young and the global disappearance of the unhappiness hump shape in age
Source: PLoS One. 2025 Aug 27;20(8):e0327858. doi: 10.1371/journal.pone.0327858 (PMC12385385; doi:10.1371/journal.pone.0327858)

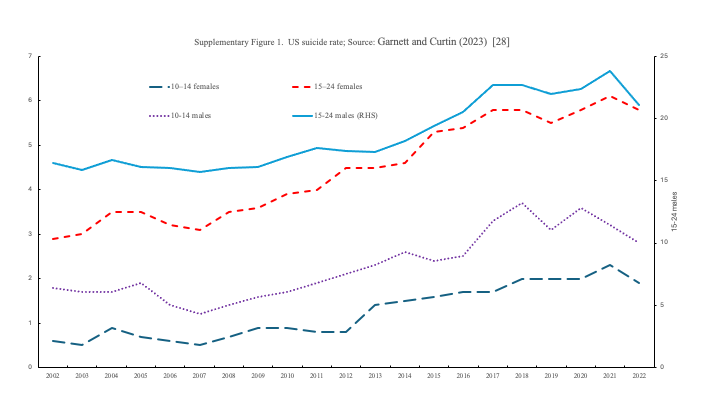

Supplement: S1 Fig — (TIF) [file pone.0327858.s001.tif]

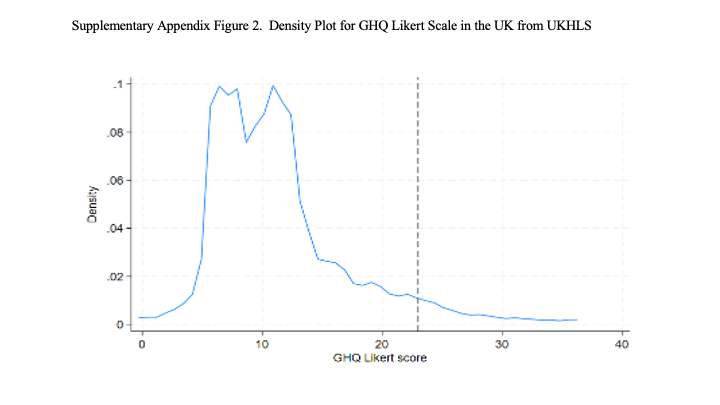

Supplement: S2 Fig — (TIF) [file pone.0327858.s002.tif]
