# Supplementary material for: The declining mental health of the young and the global disappearance of the unhappiness hump shape in age
Source: PLoS One. 2025 Aug 27;20(8):e0327858. doi: 10.1371/journal.pone.0327858 (PMC12385385; doi:10.1371/journal.pone.0327858)
Supplement: S1 Table — (DOCX) [file pone.0327858.s003.docx]

S1 Table. 44 country observations in the Global Minds database by year

2020 2021 2022 2023 2024 2025 Total

Algeria 6 7,938 20,062 14,412 9,079 5,348 56,845

Angola 12 57 4,327 5,397 4,784 1,200 15,777

Argentina 20 13,170 18,613 20,603 28,817 5,455 86,678

Australia 3,762 7,924 6,174 5,087 4,972 1,427 29,346

Bangladesh 14 32 3,140 5,190 3,194 721 12,291

Belgium 18 2,053 2,735 1,982 2,519 1,454 10,761

Bolivia 1 32 3,951 3,714 5,671 2,189 15,558

Brazil 4 94 193 260 6,335 8,236 15,122

Canada 2,703 10,912 8,257 6,916 8,388 2,561 39,737

Chile 8 2,084 4,158 6,130 5,270 1,222 18,872

Colombia 18 10,508 12,760 14,811 14,112 5,018 57,227

Ecuador 1 2,117 3,554 3,463 4,527 1,551 15,213

Egypt 5 5,064 31,105 30,863 21,043 9,463 97,543

El Salvador 0 28 4,605 3,320 2,894 1,043 11,890

France 42 3,137 13,892 13,706 9,836 3,063 43,676

Germany 57 213 8,164 8,346 7,651 3,690 28,121

Guatemala 1 2,478 5,148 5,244 2,927 1,231 17,029

Honduras 6 16 4,013 3,581 2,682 944 11,242

India 15,371 25,105 35,864 53,502 60,811 20,772 211,425

Iraq 1 2,773 13,105 8,519 3,386 5,993 33,777

Ireland 50 3,940 2,004 1,982 2,491 676 11,143

Israel 8 33 74 1,119 14,219 2,614 18,067

Italy 36 290 202 10,634 16,785 5,236 33,183

Jordan 3 55 9,835 10,430 6,467 5,366 32,156

Kenya 2 13 1,872 2,239 4,561 1,951 10,638

Mexico 20 12,847 28,652 32,373 18,811 6,394 99,097

Morocco 3 3,900 14,983 9,338 6,358 2,072 36,654

Mozambique 0 4 2,950 5,352 3,880 1,151 13,337

New Zealand 1,249 5,140 1,968 2,165 1,937 739 13,198

Nicaragua 1 10 4,105 3,132 2,718 1,103 11,069

Nigeria 4 6,498 5,944 8,553 8,278 1,571 30,848

Pakistan 13 21 13,573 20,100 13,730 3,632 51,069

Paraguay 0 8 4,738 4,198 4,519 1,456 14,919

Peru 1 3,368 8,281 9,184 5,377 2,426 28,637

Philippines 116 170 8,630 8,790 7,406 5,762 30,874

Saudi Arabia 4 1,764 5,025 5,193 2,722 1,809 16,517

South Africa 1,992 11,943 9,600 7,943 6,576 2,266 40,320

Spain 25 8,182 11,493 13,703 13,091 3,315 49,809

Tunisia 3 3,813 8,680 5,726 2,877 2,219 23,318

UK 7,206 17,497 15,259 11,149 12,038 2,281 65,430

USA 14,790 25,451 25,109 24,532 24,721 9,739 124,342

Uruguay 4 26 4,753 4,123 3,427 1,274 13,607

Venezuela 3 13,891 15,756 18,444 16,047 7,305 71,446

Yemen 0 2,757 12,136 10,533 6,695 2,569 34,690

Total 47,583 217,356 419,442 445,981 414,629 157,507 1,702,498
